# Correlations of single nucleotide polymorphisms of *CRYAA* and *CRYAB* genes with the risk and clinicopathological features of children suffering from congenital cataract

**DOI:** 10.1097/MD.0000000000007158

**Published:** 2017-06-23

**Authors:** Xian-Jin Cui, Feng-Yan Lv, Feng-Hua Li, Kun Zeng

**Affiliations:** a Department of Ophthalmology, Linyi People's Hospital; b Department of Infectious Diseases, Affiliated Hospital of Shandong Medical College, Linyi; c Shenzhen Key Laboratory of Ophthalmology, Shenzhen Eye Hospital, Shenzhen, P.R. China.

**Keywords:** clinicopathological features, congenital cataract, *CRYAA*, *CRYAB*, risk, single nucleotide polymorphism

## Abstract

**Background:**

The study aims to explore the correlations of the single nucleotide polymorphisms (SNPs) of CRYAA and CRYAB with the risk and clinicopathological features of children with congenital cataract.

**Methods:**

The study enrolled 168 children diagnosed as congenital cataract (case group) and 172 normal children (control group) from May 2015 to May 2016. Genomic DNA extraction was performed using a QIAamp DNA blood mini kit. Polymerase chain reaction (PCR) products were genotyped using an ABI direct sequencer. Haplotype, allele, and genotype frequencies of *CRYAA* and *CRYAB* gene polymorphisms analyses were carried out using the SHEsis software. Logistic regression analysis was performed in order to analyze the risk factors for children suffering from congenital cataract.

**Results:**

Presence of significant differences between the case and control groups’ genotype and allele frequencies of *CRYAA* rs7278468 and *CRYAB* rs370803064/rs387907338. TA of *CRYAB* gene might increase congenital cataract risk in children, while GCG of *CRYAA* gene and GC of *CRYAB* gene might decrease congenital cataract risk in children. *CRYAA* rs7278468, *CRYAB* rs370803064/rs387907338 polymorphisms were significantly correlated to uncorrected visual acuity, best-corrected visual acuity, nystagmus, visual axis opacification, microcornea, lens opacity, posterior capsular thickening, and degrees of posterior capsule opacification after operation in children with congenital cataract. Logistic regression analysis revealed that the T allele of *CRYAA* rs7278468, A allele of *CRYAB* rs370803064, T allele of *CRYAB* rs387907338, family history, and TA haplotype of *CRYAB* gene were risk factors for children with congenital cataract.

**Conclusion:**

Our findings demonstrated that *CRYAA* rs7278468 and *CRYAB* rs370803064/rs387907338 are correlated with the risk and clinicopathological features of children suffering from congenital cataract.

## 1 Introduction

Congenital cataract is the most common treatable cause of pediatric visual disability, due to the metabolic disorders of embryonic lens transparency during early fetal period.^[1]^ It is characterized by ocular lens opacification, which usually results from an injury to the lens micro-architecture leading to light scatter, or the formation of protein augment causing loss of transparency.^[2]^ Congenital cataract prevalence is estimated to range from 0.6 to 6 per 10,000 live births, with an incidence rate of about 2.2 to 2.49 in every 10,000 live births, and an approximate 40% cases with congenital cataract are reported to be inherited in isolation or due to ocular syndrome or abnormalities.^[3]^ Nearly a-third of congenital cataract cases are familial with an autosomal dominant or recessive inheritance.^[4]^ Despite the remarkable improvements in the clinical cataract management and updated information of lens structure and function, the correlations among cataract morphology, etiology, and mechanisms remain unclear. Accumulating reports demonstrate that genetic functions are greatly involved in the whole process.^[5,6]^ At present, most advances have been made to identify the role of genes in causing autosomal congenital cataract.^[7,8]^

Various gene mutations have been linked to congenital cataract, including structural protein genes, transcription factors, transport molecules, and cell adhesion molecules.^[9]^ Families with heritable cataract have indicated gene mutations associations with lens crystallins, among which α-crystallins are key water soluble proteins which are expressed in the lens to contribute to lens clarity maintenance.^[10]^ α-Crystallins are mainly comprised of 2 proteins, namely, αA- and αB-crystallins, at a molar ratio of 3:1,^[11]^ which are encoded by individual genes localized on disparate chromosomes, crystallin αA (*CRYAA*), and crystallin αB (*CRYAB*) in the small heat-shock protein (sHSP) family.^[12]^ They can form hetero-oligomers that bind and isolate injured proteins, inhibiting the formation of particulates that are able to scatter light.^[13]^ The *CRYAA* gene is expressed densely in the lens, while *CRYAB* is ubiquitously expressed in a broad variety of tissues and is correlated with neurologic, cardiac, and muscular dysfunctions.^[14]^ The *CRYAA* gene is mapped to chromosome 21q22.3, which consists of 3 exons.^[15]^ Located on chromosome 11q23, *CRYAB* encodes for a member of the sHSP family composing of 175 amino acid protein,^[4]^ and functions as a molecular chaperone, restraining the accumulation of denatured proteins after exposure to stresses, including radiation, heat shock, and oxidative stress.^[13]^ Currently, over 40 loci have been mapped in congenital cataract development.^[3]^ Our study targets to elucidate the effects of mutations on loci rs7278468, rs3761382, and rs13053109 of *CRYAA* and rs370803064 and rs387907338 of *CRYAB* on risks of pediatric congenital cataract to provide more genetic information on the cause of congenital cataract.

## 2 Materials and methods

### 2.1 Study subjects

The study case group included a total of 168 children (107 male and 61 female) with a calculated mean age of 5.2 ± 1.2 years, who were diagnosed with congenital cataract in Linyi People's Hospital from May 2015 to May 2016. The inclusion criteria were as follows: a diagnosis of monocular or binocular congenital cataract^[16]^; an excess of 3 mm-limit in opacification of crystallin lens, nucleus of the lens or posterior pole; patients capable of performing the Snellen visual acuity test; no complications post successful surgery and patients present with complete post-operative review; and no ocular complications like congenital glaucoma, persistent hyperplastic primary vitreous (PHPV), or retinopathy of prematurity (ROP). The exclusion criteria were as follows: patients with a history of ocular injury; congenital ocular anomaly; mentally disturbed patients; indications for surgery; and other ocular or systemic diseases. An additional 172 normal children (92 male and 80 female) with a calculated mean age of 5.3 ± 1.3 years, who underwent physical examinations, were recruited as the control group during the same period. It was ensured that the case group had no blood relations with the control group. The study was approved by the Ethics Committee of Linyi People's Hospital, and signed informed consents were obtained from all study subjects.

### 2.2 Blood sampling and DNA extraction

Fasting peripheral venous blood samples (5 mL) were collected from the case group patients within 24 hours of admission. Two percent ethylenediamine tetraacetic acid (EDTA) was added to the samples followed by preservation at −80 °C. DNA was extracted from the entire blood sample (200 μL) using a QIAamp DNA blood mini kit (Qiagen, Hilden, Germany). Purity of the extracted DNA was determined by an ultraviolet spectrophotometer with A260/A280 ratio in the range of 1.8 to 2.0. After agarose gel electrophoresis for DNA verification, the required DNA template concentration for polymerase chain reaction (PCR) was then calculated. Extracted genomic DNA was stored in the TE buffer at −80 °C prior to analyses.

### 2.3 Detection of single nucleotide polymorphisms (SNPs) in *CRYAA* and *CRYAB* genes

Genotypes of *CRYAA* and *CRYAB* genes were examined using an ABI3100-Avant sequencer. *CRYAA* and *CRYAB* gene sequences were obtained from the GenBank. PCR primers for rs7278468, rs3761382, and rs13053109 sequences in the *CRYAA* gene and rs370803064 and rs387907338 sequences in the *CRYAB* gene were designed using the Primer Premier 5.0 software (Table 1).

**Table 1:**
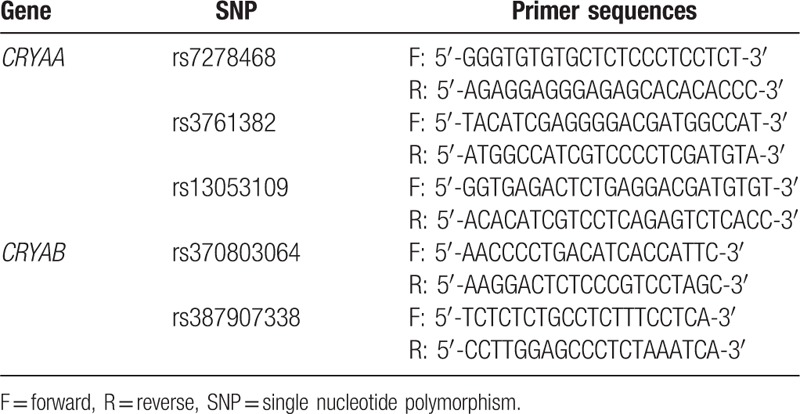
Primer sequences for *CRYAA* SNPs (rs7278468, rs3761382, and rs13053109) and *CRYAB* SNPs (rs370803064 and rs387907338).

**Figure 1:**
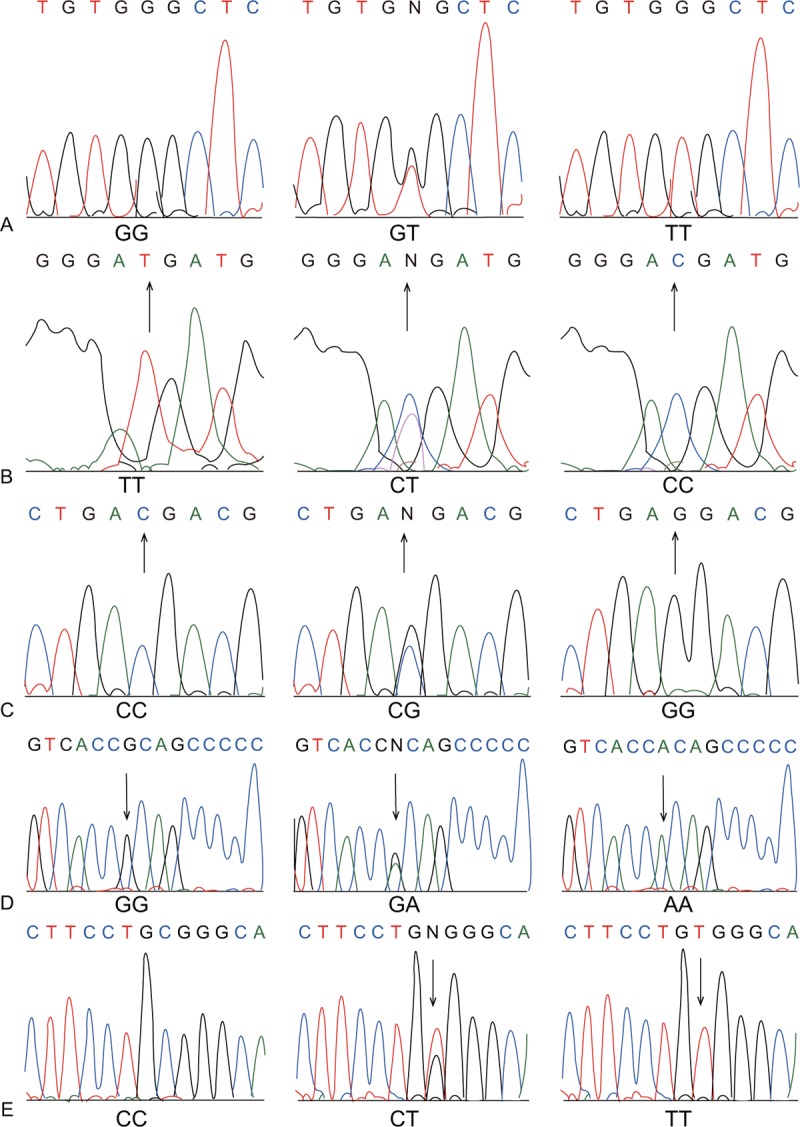
Sequencing results of *CRYAA* SNPs (rs7278468, rs3761382, and rs13053109) and *CRYAB* SNPs (rs370803064 and rs387907338). A, *CRYAA* rs7278468; B, *CRYAA* rs3761382; C, *CRYAA* rs13053109; D, *CRYAB* rs370803064; and E, *CRYAB* rs387907338. SNP  =  single nucleotide polymorphism.

The total reaction volume was 50 μL, which included 5 μL of 10 ×  buffer solution, 1 μL of DNA template (5 ng/pL), 0.75 μL of upstream primer and downstream primers each, 5 μL of dNTPs, 0.5 μL (2.5 U) of Prime STAR DNA polymerase, and 32 μL of ddH_2_O. A gradient PCR instrument was used to conduct the qRT-PCR, and amplification conditions were as follows: 3 minutes of predenaturation at 94 °C, 35 cycles of 30 seconds at 94 °C, 1 minute at 55 °C and 65 second at 72°C, and a final extension for 10 minutes at 72 °C. Two percent agarose gel electrophoresis was employed in order to purify the obtained PCR products after detection. Denaturation was performed and the sequences and genotypes were detected by an ABI3100-Avant sequencer (Applied Biosystems, Inc., CA) (Fig. 1).

### 2.4 Operative procedures and postoperative observation

Anesthesia evaluations were conducted before operations, and patients were operated on only when under the influence of general anesthesia. The same doctor conducted all procedures (sclera tunnel incision, lens extraction, posterior capsulotomy, anterior vitrectomy, and posterior chamber lens implantation) on all patients. After successful operations, patients received various routine eye examinations for uncorrected visual acuity, corrected visual acuity in mydriatic optometry, visual field, intraocular pressure, fundus, external eyes, anterior junction under slit lamp, etc. and follow-up visits. Best-corrected visual acuity was recorded based on the Snellen chart 1 month after the operation.^[17]^

Efficacy evaluations of the opacification of posterior pole in patients were made postoperation.^[18]^ The classification and evaluation criteria were as follows: 0, no opacification; 1, appearance of microfold or lens epithelial cells in posterior capsule; 2, appearance of honeycomb opacification and lens epithelial cells or fiber membrane in posterior capsule; 3, appearance of Elschnig pearl or thick fiber membrane; 4, appearance of fundus-blocking Elschnig pearl.

### 2.5 Statistical methods

Data analyses were performed using the SPSS 20.0 integrated software. Measurement data were presented as mean ± standard deviation. Comparisons between 2 groups, and comparisons among 3 groups were performed using the *t* test and one-way analysis of variance, respectively. Categorical data were shown as percentage or rate, which was further examined by a chi-square test. Odds ratio (OR) with 95% confidence interval (95% CI) was used to estimate the correlations of SNPs with children with congenital cataract. Examinations of representativeness of the population were made using the Hardy–Weinberg equilibrium. Haplotype analyses were performed by the SHEsis software, and a chi-square test was used for frequency comparison between groups. Logistic regression analysis was used to analyze the risk factors for children with congenital cataract. All tests were two-sided, with *P* < .05 was considered as statistically significant.

## 3 Results

### 3.1 Baseline characteristics of the subjects in the case and control groups

As shown in Table 2, the case and control groups revealed no remarkable differences in terms of age, gender, and state of systemic disease (all *P* > .05). The case group showed significant differences in terms of mean visual acuity, family history, and viral infection in uterus or antibiotic injection in the 1st 3-month pregnancy, metabolic disease in pregnancy in comparison with the control group (all *P* > .05).

**Table 2:**
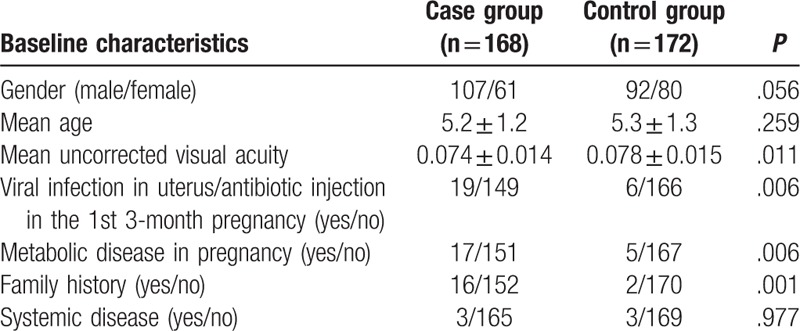
Comparisons of baseline characteristics between the case and control groups.

### 3.2 Distributions of genotype and allele frequencies of SNPs in *CRYAA* and *CRYAB* genes

The Hardy–Weinberg equilibrium demonstrated that rs7278468, rs3761382, and rs13053109 distribution in the *CRYAA* gene and rs370803064 and rs387907338 distribution in the *CRYAB* gene were consistent with the Hardy–Weinberg equilibrium (*P* > .05), further indicating that the selected groups were representative.

As shown in Table 3, the genotype and allele frequencies of rs7278468 in the *CRYAA* gene and rs370803064 and rs387907338 in the *CRYAB* gene showed evident differences between the case and control groups (all *P* < .05). The case group showed a higher frequency of T allele of rs7278468 in the *CRYAA* gene (OR  =  0.7151, 95%CI  =  0.514–0.993, *P* < .05), A allele of rs370803064 in the *CRYAB* gene (OR  =  0.7129, 95%CI  =  0.514–0.988, *P* < .05), and T allele of rs387907338 in the *CRYAB* gene (OR  =  0.6238, 95%CI  =  0.46–0.8445, *P* < .05) in comparison with the control group. However, no evident difference was identified in the frequencies of rs3761382 and rs13053109 in the *CRYAA* gene (both *P* > .05).

**Table 3:**
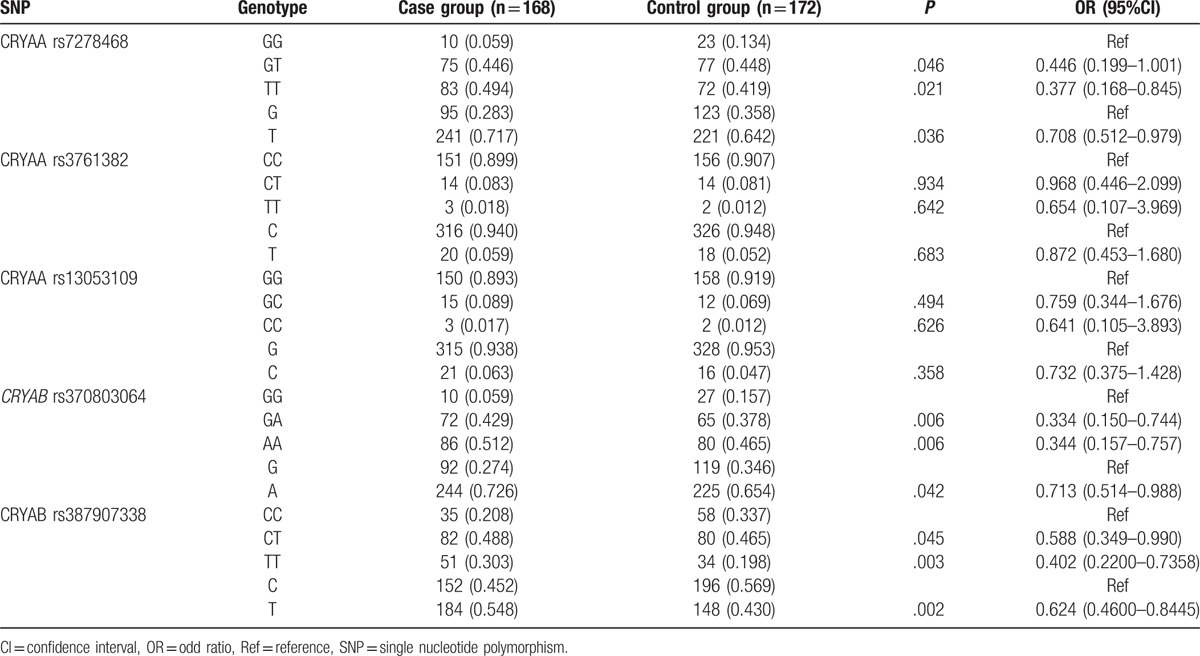
Distribution of genotypes and allele frequencies of 3 SNPs (rs7278468, rs3761382, and rs13053109) in *CRYAA* and 2 SNPs (rs370803064 and rs387907338) in *CRYAB.*

### 3.3 Haplotype analyses for SNPs in *CRYAA* and *CRYAB* genes

Haplotype analyses for rs7278468, rs3761382, and rs13053109 sequences in the *CRYAA* gene and rs370803064 and rs387907338 sequences in the *CRYAB* gene were feasible owing to the presence of a strong linkage disequilibrium among these 5 SNPs (*r*^2^ > 0.8) and those with minor allele frequency (MAF) more than 0.1 were further analyzed. The results indicated that haplotype GCG of the *CRYAA* gene might decrease congenital cataract risk for in newborn infants (OR  =  0.705, 95%CI  =  0.51–0.975, *P* < .05). TA of the *CRYAB* gene might increase congenital cataract risk, on the other hand GC might decrease congenital cataract risk in newborn infants (TA: OR  =  1.603, 95%CI  =  1.184–2.17, *P* < .05; GC: OR  =  0.713, 95%CI  =  0.514–0.989, *P* < .05) (Tables 4 and 5).

**Table 4:**
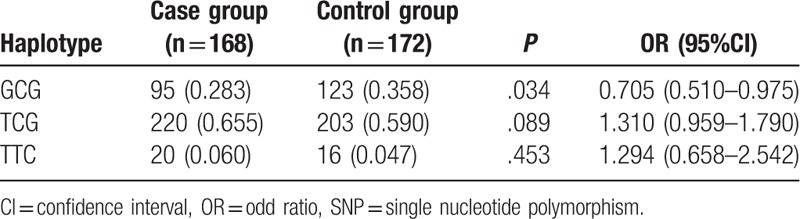
Haplotype analyses for 3 SNPs (rs7278468, rs3761382, and rs13053109) in *CRYAA.*

**Table 5:**
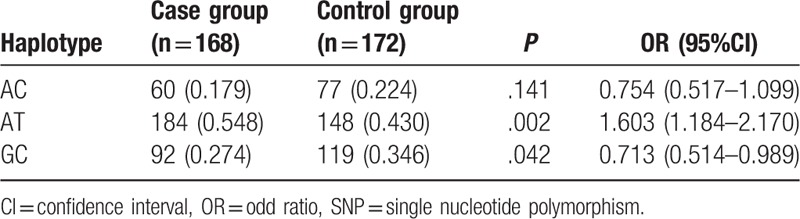
Haplotype analyses of 2 SNPs (rs370803064 and rs387907338) in *CRYAB.*

### 3.4 Relation between the clinicopathological features of children with congenital cataract and SNPs in *CRYAA* and *CRYAB* genes

As shown in Tables 6 and 7, patients presenting with a T allele in *CRYAA* rs7278468 (GT + TT) and *CRYAB* rs387907338 (TT + TC), and an A allele in *CRYAB* rs370803064 (AA + GA), respectively, showed a weaker uncorrected visual acuity and the best-corrected visual acuity and severer nystagmus, visual axis opacification, microcornea deformity, lens opacity, posterior capsular thickening, and degrees of posterior capsule opacification after the operation than the patients presenting with corresponding homozygote GG, CC, and GG (all *P* < .05).

**Table 6:**
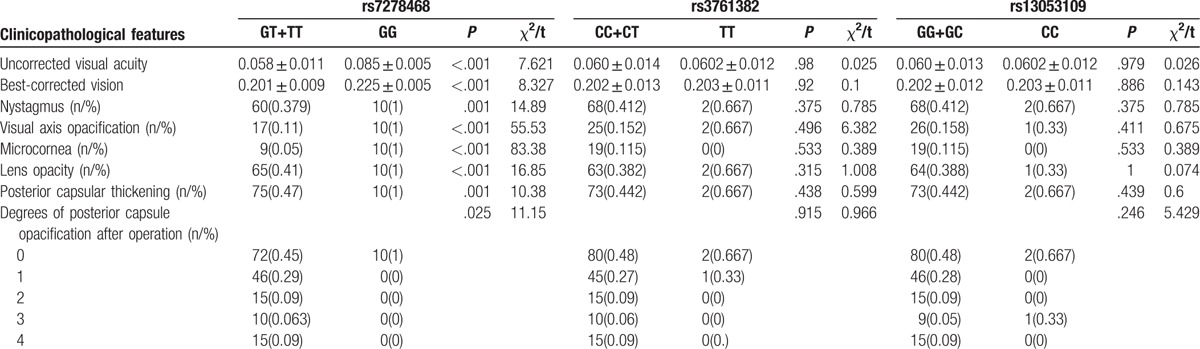
Relation between the clinicopathological features of children with congenital cataract and three SNPs (rs7278468, rs3761382 and rs13053109) in *CRYAA.*

**Table 7:**
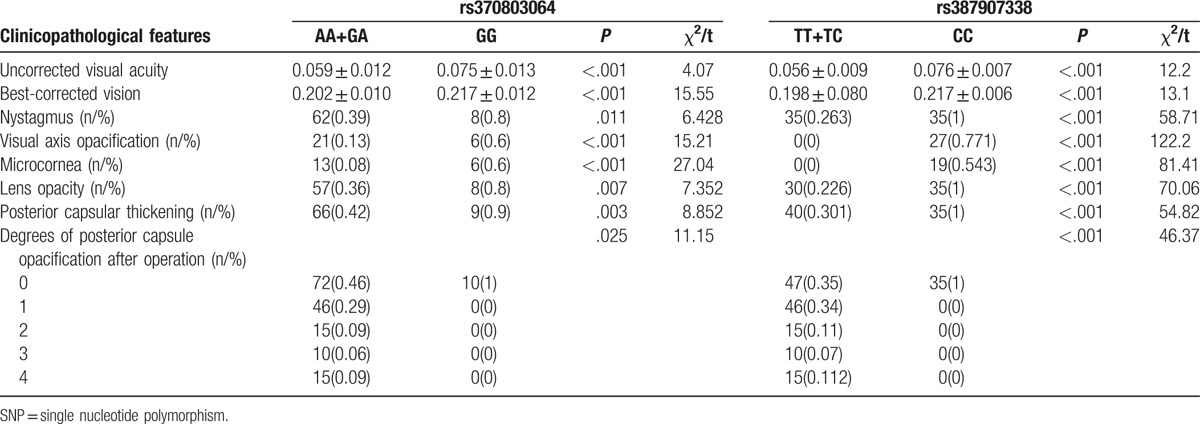
Relation between the clinicopathological features of congenital cataract and two SNPs (rs370803064 and rs387907338) in *CRYAB.*

### 3.5 Logistic regression analysis of the risk factors for children with congenital cataract

Logistic regression analyses were performed taking congenital cataract development as the dependent variable and furthermore, mean visual acuity, family history, viral infection in the uterus or antibiotic injection in the 1st 3-month pregnancy, metabolic disease in pregnancy, *CRYAA* rs7278468, *CRYAB* rs370803064 rs387907338, GCG, TA, and GC were chosen as the independent variables. The results revealed that the presence of a T allele in *CRYAA* rs7278468, A allele in *CRYAB* rs370803064, T allele in *CRYAB* rs387907338, family history, and TA are the risk factors for children suffering from congenital cataract (all *P* < .05) (Table 8).

**Table 8:**
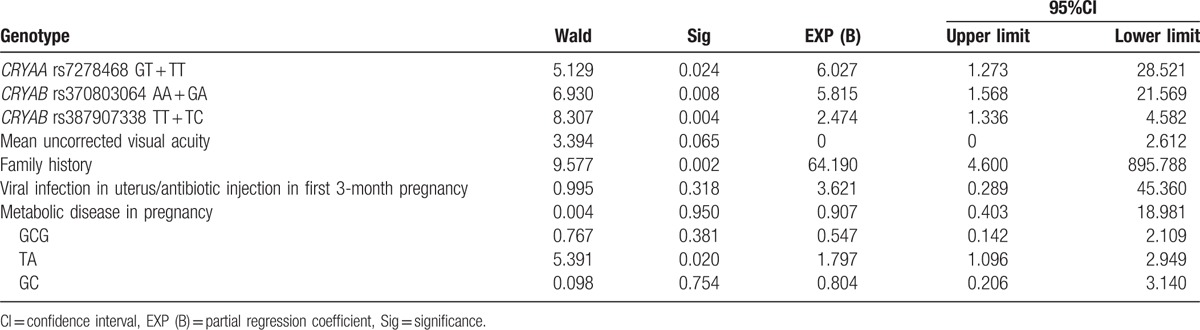
Logistic regression analysis of the risk factors for children with congenital cataract.

## 4 Discussion

*CRYAA* and *CRYAB* mutations have been reported to play a critical role in congenital cataract.^[13,19]^ Currently, over 40 loci have been mapped in congenital cataract development.^[3]^ However, the mechanism of *CRYAA* and *CRYAB* mutations leading to congenital cataract remains unclear. The present study tried to investigate the effects of mutations on loci rs7278468, rs3761382, and rs13053109 on the *CRYAA* gene and rs370803064 and rs387907338 on the *CRYAB* gene on risks of pediatric congenital cataract. Consequently, the study indicated that *CRYAA* rs7278468, *CRYAB* rs370803064, and *CRYAB* rs387907338 were associated with the risk and clinicopathological features of children with congenital cataract.

Initially, the research findings revealed a significant difference between the case and control groups’ genotype and allele frequencies of *CRYAA* rs7278468, *CRYAB* rs370803064, and *CRYAB* rs387907338. Being members of the sHSP family, *CRYAA* and *CRYAB* genes can function as molecular chaperone-like agents and accumulate related proteins in large soluble gatherings of about 30 to 40 subunits.^[1]^ The *CRYAA* gene is expressed at a higher concentration in the lens and critically participates in lens clarity maintenance, thus preventing opacification.^[11]^*CRYAA* gene is not highly expressed in the lens epithelium, whereas it shows an elevated expression in the elongation zone and plays a significant role in the differentiation from epithelial cells to fiber cells in the lens.^[20]^*CRYAB* gene mutations can result in diverse clinical phenotypes, such as isolated cataract, myopathy, myofibrillar, cardiomyopathy, as well as a multisystemic disorder with a combination of these features.^[13]^ To date, a number of mutations in *CRYAA* and *CRYAB* genes have been identified in patients or families with a congenital cataract background.^[13,20–22]^ For example, c.246_248delCGC (p.117delR), a novel mutation of the *CRYAA* gene, has been detected in a Chinese family with perinuclear congenital cataracts of autosomal type.^[15]^ Su et al^[23]^ also identified a disease-causing mutation in the *CRYAA* gene, c.161G > C (p.R54P), with autosomal dominant Y-suture cataracts. Jiaox et al^[4]^ have reported 2 novel missense mutations, namely p.R11C and p. R12C of the *CRYAB* gene, show relations with autosomal recessive congenital nuclear cataracts. It is also identified that c.59C > G (P20R) in the *CRYAB* gene was a mutant in a 5-generation family with a hereditary posterior polar cataract background.^[13]^ Moreover, evidence identified a novel missense R11H mutation of the *CRYAB* gene to be correlated with congenital nuclear cataract in a 4-generation family.^[11]^ Ma et al^[24]^ confirmed that the T allele on rs7278468 is capable of contributing to the increased frequency in patients with age related cataract (ARC), which is consistent with our results. We further indicated that *CRYAA* rs7278468, *CRYAB* rs370803064, and *CRYAB* rs387907338 show correlations with uncorrected visual acuity, best-corrected visual acuity, nystagmus, visual axis opacification, microcornea, lens opacity, posterior capsular thickening, and degrees of posterior capsule opacification after operation.

Furthermore, our study data indicated that TA of the *CRYAB* gene might increase congenital cataract risk in children, while GCG of the *CRYAA* gene and GC of the *CRYAB* gene might decrease congenital cataract risk in children. Ma et al^[24]^ found that the C-G-T haplotype could function as a risk factor for ARC, yet the T-C-G haplotype appeared to be an ARC protective factor, and the rs7278468 T allele on the *CRYAA* gene was responsible for a decrease in the transcriptional activity imparted by the original risk haplotype, and the T allele can increase its binding affinity in KLF10's binding motif, reducing *CRYAA* transcription and αA-crystallin protein levels. Logistic regression analysis also confirmed that T allele of *CRYAA* rs7278468, A allele of *CRYAB* rs370803064, T allele of *CRYAB* rs387907338, family history, and TA haplotype of *CRYAB* were risk factors for children suffering from congenital cataract.

In summary, the present study provided evidence of risk factors on the congenital cataract genetic background. *CRYAA* rs7278468, *CRYAB* rs370803064, and *CRYAB* rs387907338 were associated with the risks and clinicopathological features of children with congenital cataract. However, the etiology and pathogenesis of hereditary congenital cataract is complicated and affected by multiple factors. More researches are required to further investigate the relationship between congenital cataract and *CRYAA* and *CRYAB* genes.

## Acknowledgments

The authors thank all participants enrolled in the present study.
